# Pomegranate (*Punica granatum*) Peel Inhibits the *In Vitro* and *In Vivo* Growth of Piroplasm Parasites

**DOI:** 10.1155/2022/8574541

**Published:** 2022-06-20

**Authors:** Rasha Eltaysh, Mohamed Abdo Rizk, Bassem Elmishmishy, Shimaa Abd El-Salam El-Sayed, Khaled Abouelnasr, Ikuo Igarashi

**Affiliations:** ^1^National Research Center for Protozoan Diseases, Obihiro University of Agriculture and Veterinary Medicine, Inada-Cho, Obihiro, Hokkaido 080-8555, Japan; ^2^Department of Pharmacology, Faculty of Veterinary Medicine, Mansoura University, Mansoura, Dakahlia, Egypt; ^3^Department of Internal Medicine and Infectious Diseases, Faculty of Veterinary Medicine, Mansoura University, Mansoura 35516, Egypt; ^4^Department of Parasitology, Faculty of Veterinary Medicine, Mansoura University, Mansoura 35516, Egypt; ^5^Department of Biochemistry and Chemistry of Nutrition, Faculty of Veterinary Medicine, Mansoura University, Mansoura 35516, Egypt; ^6^Department of Surgery, Anesthesiology and Radiology, Faculty of Veterinary Medicine, Mansoura University, Mansoura 35516, Egypt

## Abstract

Pomegranate (*Punica granatum*) peel has seen a rapid surge in attention as a medical and nutritional product over the last decade. The impact of pomegranate peel methanolic extract monotherapy and combination therapy on the *in vitro* growth of *Babesia* (*B.*) *bovis*, *B. bigemina*, *B. divergens*, *B. caballi*, and *Theileria* (*T.*) *equi*, as well as *B. microti* in mice, was investigated in this work. Fluorescence-based SYBR green I assay was used for evaluating the inhibitory antibabesial efficacy of pomegranate (*Punica granatum*) peel against the growth of several piroplasm parasites *in vitro* and *in vivo*. Celltac *α* MEK-6450 computerized haematology analyzer was used for monitoring the haematological parameters of treated mice every 4 days. Pomegranate peel inhibited the *in vitro* growth of *B. bovis*, *B. bigemina*, *B. divergens*, *T. equi*, and *B. caballi* in a dose-dependent manner, with IC_50_ values of 154.45 ± 23.11, 40.90 ± 9.35, 72.71 ± 14.77, 100 ± 16.20, and 77.27 ± 16.94 *μ*g/ml, respectively. On a *B. bovis* culture, the *in vitro* inhibitory effect of pomegranate peel was amplified when it was combined with diminazene aceturate (DA). Combination therapy of pomegranate peel and a low dose of DA (15 mg kg^−1^) inhibited *B. microti* growth significantly (*P* < 0.05) higher than the treatment with the full dose of DA (25 mg kg^−1^) in *B. microti*-infected mice. These findings suggest that pomegranate peel might be a potential medicinal plant for babesiosis treatment, especially when combined with a low dosage of DA.

## 1. Introduction

Tick-borne parasites like *Babesia* and *Theileria* infect the erythrocytes of animals, inflicting major economic losses in the livestock sector and animal trade across the world [1]. Fever, malaise, jaundice, hemoglobinuria, and mortality are among the symptoms of the disease [2]. The infection is caused mostly by *Babesia (B.) bovis* and *B. bigemina* in ruminant, and by *Theileria equi (T. equi)* and *B. caballi* in equine [2]. Evaluation of the antibabesial effect of the newly developed drugs in large animals under filed condition is very difficult. Alternatively, determination the drugs effect *in vivo* is performed in a laboratory experimental animal. For this purpose, a rodent *Babesia* model infected with *B. microti* is used [3].

Presence of drug residue in the meat of the treated animals is reported after the treatment with the currently used antibabesial drug, imidocarb dipropionate [1]. In the same time, recent study detected a resistance from the treated *Babesia* parasite with another commonly used antibabesial drug, diminazene aceturate (DA) [4]. As a result, finding more effective and safer antipiroplasm agents becomes a necessity, so natural phytochemicals might be an alternative strategy. Accordingly, pomegranate (*Punica granatum)*, a mystical and highly distinctive fruit, has been used as a distinct remedy in traditional medicine for centuries to alleviate a variety of diseases [5]. *Punica granatum L.* is now a fruit that not only piques the public's attention but also stimulates study into its medical characteristics and food industry. Pomegranate has a wide range of potential therapeutic characteristics, including the treatment and prevention of cancer, cardiovascular disease, diabetes, Alzheimer's disease, arthritis, and obesity [5]. Fruit peel, seeds, flowers, the bark of young shoots and roots, leaves, and pomegranate sauce have been traditionally practicable [5]. Pomegranate peel extract (PPE) has been proven in studies to have antibacterial and antifungal [6], antimalarial [7], antiprotozoal [8], antihelminthic, and antioxidant activities [9]. The antibabesial efficacy of pomegranate (*Punica granatum*) peel extract still has to be determined. As a result, in the current investigation, we investigated the potential of pomegranate peel as an antipiroplasm candidate *in vitro* against the growth of bovine and equine *piroplasm* parasites, as well as *B. microti* in mice.

## 2. Materials and Methods

### 2.1. In Vitro Growth Inhibition Assay

Pomegranate peel powder was purchased from iherb.com and dissolved in 99.8% methanol (Wako Pure Chemical Industries, Ltd., Osaka, Japan) and prepared as previously detailed in our study [10]. Briefly, the pomegranate peel powder (10 g) was dissolved in 50 ml methanol and incubated at 30°C for three days. After that, the final product was filtered using Whatman filter paper No. 1. Using a rotary evaporator (BUCHI® Rotavapor R-200/205, Flawil, Switzerland) under reduced pressure at 40°C and a freeze-dry vacuum system, the extract was concentrated and lyophilized (Labconco, Kansas, MO, USA). The toxicity of pomegranate peel methanolic extract to bovine and equine erythrocytes was evaluated using 25 mg/ml as previously described [11].

Pomegranate peel was evaluated for its chemotherapeutic effect against *B. bovis* (Texas strain) [12], *B. bigemina* (Argentina strain) [13], *B. divergens* (German strain) [14], *B. caballi* (El-Sayed et al. 2015), and *T. equi* (U.S. Department of Agriculture) [15] using fluorescence assay using a nucleic acid stain SYBR Green I (Lonza, Rockland, USA) [12, 16]. Using a microaerophilic, stationary-phase culture method, the parasites were cultured in purified bovine or equine red blood cells (RBCs). Briefly, *B. bovis*, *B. bigemina*, and *T. equi* were cultured in Medium 199, whereas *B. caballi* was cultured in RPMI 1640 (both from Sigma-Aldrich, Japan). 40% normal bovine serum (for bovine *Babesia* isolates) or 40% normal horse serum (for equine *Babesia* and *Theileria* isolates) was added to the media, along with 60 U/ml penicillin G, 60 *μ*g/ml streptomycin, and 0.15 *μ*g/ml amphotericin B (all three drugs from Sigma-Aldrich). A critical supplement of 13.6 *μ*g hypoxanthine (ICN Biomedicals, Inc., USA) per ml was also supplied to the *T. equi* culture. Cultures of parasitized RBCs (pRBCs) were incubated at 37°C in an atmosphere of 5% CO_2_, 5% O_2_, and 90% N_2_. Pomegranate peel concentrations ranged from 0.025 to 30 mg/ml was used. The commonly used antibabesial drug, DA, was used as a controlled drug for the *in vitro* study [17, 18]. Cultures of pRBCs without the medication and cultures containing only DMSO were generated as negative experimental controls. Nonparasitized bovine or equine RBCs were used as a blank control. The parasite *in vitro* regrowth after stopping the treatment by pomegranate peel was monitored for all screened parasites using viability assay as previously detailed in our study [14]. All experiments were repeated three times.

### 2.2. Pomegranate Peel and DA Combination Test

Combination therapies of pomegranate peel with DA was tested against the *in vitro* cultures of bovine *Babesia*, and equine *piroplasm* parasites exhibited the highest IC_50_s values, *B. bovis* and *T. equi* following the method previously detailed in our study [3]. The experiments were repeated three times.

### 2.3. In Vivo Inhibitory Effect of Pomegranate Peel in Mice

The pomegranate peel *in vivo* inhibition assay for *B. microti* (Munich strain) [19] in 25 female BALB/c mice aged 8 weeks and weighted 18-20 gm (CLEA Japan, Tokyo, Japan) was performed twice using fluorescence assay [20]. Five groups of mice were used (five mice/group). All mice were intraperitoneally infected with 1 × 10^7^*B. microti*-RBCs using the equation (parasitemia/1000) × counted RBCs × 200 × 5 × 10^4^, whereas parasitemia/1000 is the parasitemia in donor mice counted in 1000 RBCs, counted RBCs is the number of RBCs counted using hemocytometer, 200 is the times of dilutions, 5 is the number of counted squares using hemocytometer, and 104 to get the parasitemia in 1 ml. The level of parasitemia in the infected mouse was monitored daily using Giemsa-stained thin blood smears prepared from venous tail blood, and when the parasitemia reached approximately 1% (with range 0.98-1.1%), the treatment was started and continued for 5 successive days. In control group, mice were injected with I/P doses of DMSO in phosphate-buffered saline (PBS) (0.02%). DA and pomegranate (*Punica granatum*) peel were administrated in subcutaneous and oral dosages, respectively. Pomegranate peel was administered at a dose rate 75 mg/kg. Drugs in the combination therapy was administrated in the same inoculation time at dose rates 50 mg/kg pomegranate (*Punica granatum*) peel and 15 mg/kg DA. Simultaneous with evaluation of the drug inhibitory effect, 10 *μ*l of blood was collected from the tail of each mouse for monitoring the haematological parameters every 4 days using Celltac *α* MEK-6450 computerized haematology analyzer (Nihon Kohden Corporation, Tokyo, Japan).

### 2.4. Statistical Analysis

The obtained data were subjected to statistical analysis using GraphPad Prism (Software, Inc., San Diego, CA, USA). A one-way ANOVA test is used for determination of the statistical significant between treated and nontreated groups. All obtained values are considered statistically significant when *P* < 0.05.

## 3. Results

### 3.1. Pomegranate Peel a Promising Candidate for Treatment of *B. bigemina* and *B. divergens*

**Figure 1:**
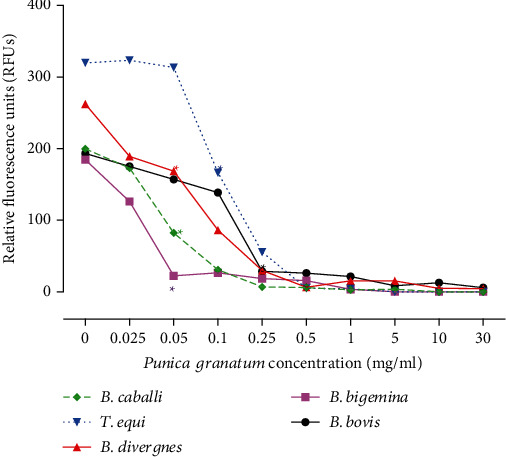
Inhibitory effect of pomegranate (*Punica granatum*) peel on *B. bovis*, *B. bigemina*, *B. divergens*, *T. equi*, and *B. caballi* on the fourth day of treatment. Each value represents the mean of triplicate trials after subtraction of the background fluorescence for nonparasitized RBCs. Asterisks denote a significant difference between the pomegranate (*Punica granatum*) peel-treated and control cultures (*P* < 0.05).

The *in vitro* growths of *B. bigemina* and *B. divergens* were greatly inhibited by pomegranate peel followed by *B. caballi* (Table 1). The *in vitro* growth of *B. divergens*, *B. bigemina*, and *B. caballi* was significantly inhibited (*P* < 0.05) by 0.05 mg/ml pomegranate peel (Figure 1). Furthermore, pomegranate peel treatments of 0.10 and 0.25 mg/ml significantly suppressed (*P* < 0.05) the development of *T. equi* and *B. bovis*, respectively (Figure 1). In the subsequent viability test, *B. bigemina, B. divergens*, and *B. caballi in vitro* regrowths were suppressed at a dosage of 0.1 mg/ml (Table 2). *In vitro* treatment of *T. equi* with 0.25 mg/ml pomegranate peel inhibited parasite regrowth (Table 2). *B. bovis* regrowth was started to be suppressed at 0.5 mg/ml pomegranate peel (Table 2). DA prevented the piroplasm *in vitro* regrowth even at 0.25 *μ*M (Table S1). The lack of a significant difference (*P* > 0.05) between the DMSO-treated positive control well and the untreated wells demonstrates that the diluent had no effect on the pomegranate peel methanolic extract's efficacy. Moreover, pretreatment of erythrocytes with a high dose of pomegranate peel methanolic extract 25 mg/ml had no effect on parasite growth pattern or erythrocyte morphology when compared to nontreated erythrocytes (data not shown).

### 3.2. DA Enhanced the In Vitro and In Vivo Efficacy of Pomegranate Peel Methanolic Extract

Different combinations of pomegranate peel with DA on *B. bovis* and *T. equi* have been evaluated. A high concentration of pomegranate peel (0.75) revealed synergistic interaction with all used concentrations of DA on the growth of *B. bovis* (Table 3), while low concentration of pomegranate peel exhibited additive interaction with DA against the growth of bovine *Babesia* parasite (Table 3). Treatments of *B. T. equi* parasite with pomegranate peel/DA combined therapy revealed indifferent interaction for all used concentrations (Table 3). Such findings confirmed the potential anti-*B. bovis* effect of pomegranate peel, especially when administrated in high doses simultaneously with the commonly used antibabesial drug, DA.

**Figure 2:**
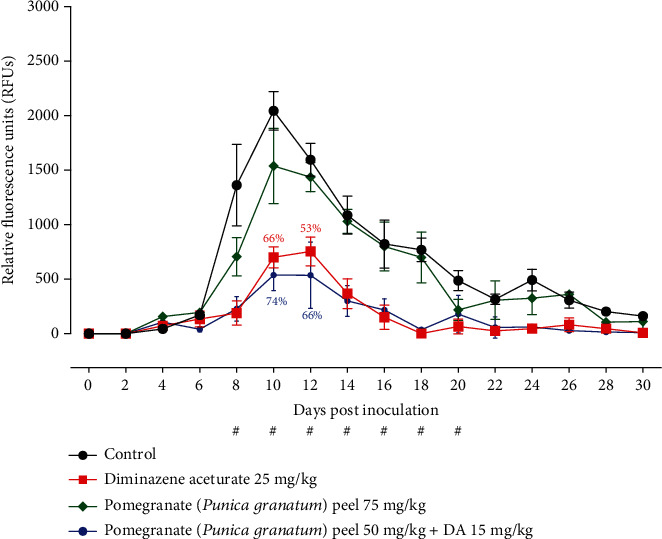
Inhibitory effect of pomegranate (*Punica granatum*) peel mono- and combination therapies on the growth of *Babesia microti* in BALB/C mice. The values are presented as mean and SD of the used mice in each group. # indicates significant differences (*P* < 0.05) between either the pomegranate peel/DA or DA monotherapy-treated and control groups.

The *in vivo* inhibitory effect of pomegranate peel was evaluated against *B. microti* in mice. After days 8 to 20 p.i., pomegranate peel-treated mice showed significant suppression (*P* < 0.05) in the fluorescence values when compared to the positive control group (Figure 2 and Table S2). The highest fluorescence values within the pomegranate peel-treated groups reached a mean of 1538.61, 537.71 within the presence of 75 mg kg^−1^ pomegranate peel monotherapy, and 50 mg kg^−1^ pomegranate peel with 15 mg kg^−1^ DA at 10 days p.i., respectively (Figure 2 and Table S2). In contrast, within the positive control group and 25 mg kg^−1^ DA monotherapy, the peak fluorescence values were 2044.96 and 754.66 at 10 and 12 days p.i., respectively (Figure 2 and Table S2). At 10 and 12 days postinjection, the inhibition within the fluorescence values was larger in mice treated with pomegranate peel/DA combination than in animals treated with 25 mg kg^−1^ DA (Figure 2 and Table S2). Oral injections of 50 mg kg^−1^ pomegranate peel, when administrated in combination with subcutaneous dose 15 mg kg^−1^ DA, caused 74% and 66% inhibition rates within the parasite growth at 10 and 12 days pi, respectively, compared with 66% and 53% inhibitions within the presence of 25 mg kg^−1^ DA at days 10 and 12 pi (Figure 2 and Table S2).

**Figure 3:**
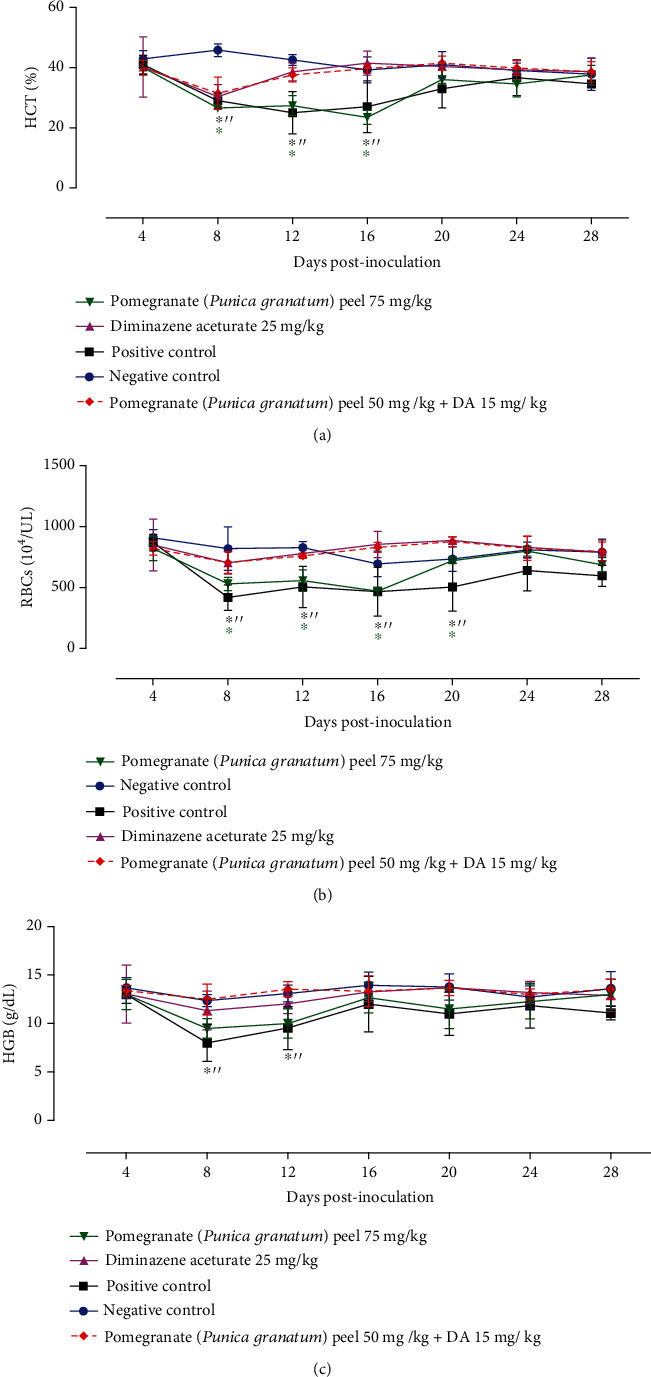
Hematological variables in *B. microti*-infected mice treated with pomegranate (*Punica granatum*) peel. (a) Hematocrit (HCT). (b) RBCs. (c) Hemoglobin (HGB). The values are presented as mean and SD of the mice used in each group. The significant difference (*P* < 0.05) between the used groups is indicated by asterisks.

Treatment of mice with pomegranate peel/a low dose of DA normalized the assessed hematological variables similar with those treated with 25 mg kg^−1^ DA (Figure 3). This result confirms the potential antibabesial efficacy of pomegranate peel/DA. A nested PCR targeting the *B. microti ss-rRNA* gene was performed on day 30 p.i. to detect parasite remnants in the blood and various organs of treated mice. Unfortunately, the parasite gene was detected in the blood and body tissues of mice treated with either DA monotherapy or pomegranate peel/DA combination therapy (Figure S1).

## 4. Discussion

This study investigated that pomegranate peel inhibited the growth of *B. bovis*, *B. bigemina*, *B. divergens*, *T. equi*, and *B. caballi in vitro* and *B. microti in vivo.* For the chosen *Babesia* species, pomegranate peel had lower IC50 values than *allicin*, a well-known herbal antibabesial medicine [21], turmeric (*Curcuma longa*) [17], and *Zingiber officinale* rhizome [10].

Interestingly, that pomegranate's therapeutic effects on splenic damage and oxidative stress in *Plasmodium chabuadi*-infected mice have been described, indicating the potential effectiveness of pomegranate as an antioxidant, antimalarial, and anti-inflammatory drug [7]. The antibabesial activity of pomegranate may be explained by the biological similarities between *Plasmodium* and *Babesia* parasites. Taken simultaneously, pomegranate's high-antioxidant action may explain the antibabesial potency of this medicinal plant, as *Babesia* infection is often accompanied by an increase in free radicals and oxidative stress biomarkers which is harmful to the infected host [22]. Keeping in mind, previous studies [23, 24] reported the richness of pomegranate peel by *gallic acid* and *quercetin*, and at the same time, the potent antibabesial efficacy of these two components [25] was reported. Therefore, the presence of these bioactive compounds in pomegranate peel might explain why this medicinal plant has antibabesial properties. Of note, in the current investigation, very high amounts of pomegranate peel had no adverse effect on bovine or horse RBCs. This justifies why pomegranate and its constituents have safely been consumed long ago. These findings back up the nontoxic effect of pomegranate peel and confirm the safety of pomegranate peel *in vitro* trials and suggest that this promising antipiroplasm candidate be tested *in vivo*. Studies in animals reveal that concentrations of pomegranate and its constituents routinely used in folk and traditional medicine note no toxic effects [26]. Cerdá et al. [27] reported that polyphenol antioxidant *punicalagin*, abundant in pomegranate juice, exhibit no toxic effects on rat organs which were examined using histopathological tests. Even in the human aspect, previous trials proved the safety of a tableted pomegranate at doses up to 1,420 mg/day (870 mg gallic acid equivalents) for 28 days, with no reported side effects or changes in blood or urine laboratory values [28].

The *in vitro* inhibitory effect of pomegranate peel together with the safety of pomegranate peel motivates us to further investigate the inhibitory effect of pomegranate peel when used either alone or in combination with DA in mice. The *in vitro* inhibitory effect of pomegranate peel was enhanced in our study when used in combination with DA against the growth of *B. bovis.* These results are similar to the *in vitro* inhibitory effects of *myrrh oil*/DA [11], *allicin*/DA [21], and *TQ*/DA combinations [19]. For *in vivo* study, the inhibition in *B. microti* growth caused by pomegranate peel/DA is higher than 70% inhibition rates for clindamycin combined with the natural product quinine [29], 56.35 and 53.25% inhibition rates for 85 mg kg^−1^ PYR combined with 10 mg kg^−1^ DA [20], 67% inhibition rates for 50 mg kg^–1^ enoxacin and 10 mg kg^−1^ DA [3], and 62.5% inhibition rates for 50 mg kg^–1^ oral dose of *TQ* and 10 mg kg^−1^ subcutaneous dose of DA [19].

The efficacy of the administered combination treatment to eliminate parasite nucleic acid from the animal's body was tested using a nested PCR assay. Combination therapy was not effective in removing parasite nucleic acid from the blood and the tested body tissues of treated mice. The same results were observed after treatment of the mice by PYR/DA combination on day 30 p.i. [20]. The short period of the experiment may explain the presence of such remnant.

## 5. Conclusions

*B. bigemina* and *B. divergens* preceded by *B. caballi* were the most susceptible Babesia species to the in vitro inhibitory action of pomegranate peel. Pomegranate peel in combination with DA exhibited synergistic interaction against the *in vitro* growth of *B. bovis.* Mice treated with a combination therapy containing lower dosages of pomegranate peel and DA showed a considerable reduction in fluorescence levels. Furthermore, the pomegranate peel/DA combination therapy normalizes the haematological variables and treat hemolytic anemia associated with babesiosis. According to the findings of this study, pomegranate peel may be effective in the treatment of animal piroplasmosis, particularly when combined with DA. Although this study evaluated the antibabesial efficacy of methanolic extract of pomegranate peel *in vitro* and *in vivo*, the study neglected the identification of the medicinally active substances in the methanolic extract of pomegranate peel. Therefore, future studies are required to perform phytochemical analysis to the used extract. Also, further studies are required to examine the ability of pomegranate peel/DA to remove the nucleic acid of the parasite from different body tissues of the treated mice after prolonged period of the experiment.

## Figures and Tables

**Table 1:** IC_50_ values of pomegranate (*Punica granatum*) peel, diminazene aceturate, and other previously used herbal antibabesial drugs evaluated for bovine *Babesia* and equine *Babesia* and *Theileria* parasites.

Organism	IC_50_ (*μ*g/ml)^a^			
	*Punica granatum*	Diminazene aceturate	*Zingiber officinale* rhizome^b^	Turmeric (*Curcuma longa*)^c^
*B. bovis*	154.45 ± 23.11	0.16 ± 0.02	588 ± 23.80	830 ± 78
*B. bigemina*	40.90 ± 9.35	0.08 ± 0.003	14800 ± 1240	ND
*B. divergens*	72.71 ± 14.77	0.046 ± 0.007	ND	375 ± 55
*T. equi*	100 ± 16.20	0.28 ± 0.01	39350 ± 1340	1405 ± 575
*B. caballi*	77.27 ± 16.94	0.012 ± 0.003	356.05 ± 34.71	720 ± 90

^a^IC_50_ values for pomegranate (*Punica granatum)* peel and diminazene aceturate were calculated on the fourth day based on the growth inhibitions determined using fluorescence-based assay in three separate experiments. Each drug concentration was made in triplicate in each experiment, and the final obtained IC_50_ represents the mean and standard deviation of three separate experiments. ND: not detected. ^b^The IC_50_ was reported in the previous study [10]. ^c^The IC_50_ was reported in the previous study [17].

**Table 2:** Viability test results of pomegranate (*Punica granatum*) peel evaluated for *Babesia* and *Theileria* parasite.

Drug	Drug concentrations (mg/ml)^a^								
	0.025	0.05	0.1	0.25	0.5	1	5	10	30
*B. bovis*	+	+	+	+	-	-	-	-	-
*B. bigemina*	+	+	-	-	-	-	-	-	-
*B. divergens*	+	+	-	-	-	-	-	-	-
*T. equi*	+	+	+	-	-	-	-		-
*B. Caballi*	+	+	-	-	-	-	-	-	-

^a^Each value was calculated using fluorescence assay in three separate experiments. Each concentration of the drug was made in triplicate in each experiment. +: viable; -: dead.

**Table 3:** Two drug interactions of pomegranate (*Punica granatum*) peel in combination with diminazene aceturate on the *in vitro* growth of *Babesia bovis* and *Theileria equi* parasites.

Parasite	*C* ^a^	FIC_D1_	FIC_D2_	*Σ*FIC	Degree of interaction^b^
*B. bovis*	0.75 + 0.75	0.33	0.01	0.34	Synergetic
	0.75 + 0.50	0.21	0.12	0.33	Synergetic
	0.75 + 0.25	0.11	0.23	0.34	Synergetic
	0.50 + 0.75	0.21	0.45	0.66	Additive
	0.50 + 0.50	0.11	0.44	0.55	Additive
	0.50 + 0.25	0.23	0.45	0.68	Additive
	0.25 + 0.75	0.23	0.61	0.84	Additive
	0.25 + 0.50	0.45	0.23	0.68	Additive
	0.25 + 0.25	0.32	0.56	0.88	Additive

*T. equi*	0.75 + 0.75	0.45	0.78	1.23	Indifferent
	0.75 + 0.50	0.77	0.56	1.33	Indifferent
	0.75 + 0.25	0.75	0.45	1.2	Indifferent
	0.50 + 0.75	0.88	0.53	1.41	Indifferent
	0.50 + 0.50	0.88	0.23	1.11	Indifferent
	0.50 + 0.25	0.98	0.98	1.96	Indifferent
	0.25 + 0.75	1.45	0.23	1.68	Indifferent
	0.25 + 0.50	0.88	0.87	1.75	Indifferent
	0.25 + 0.25	1.02	0.78	1.80	Indifferent

^a^ *C* refers to the different concentrations of pomegranate (*Punica granatum*) peel in combination with diminazene aceturate. ^b^The degree of drug interaction was determined based on the following fractional inhibitory concentration (FIC) index: ≤0.5 (synergetic), >0.5–1 (additive), and >1 to <2 (indifferent). FIC_D1_ refers to the fractional inhibitory concentration of *Punica granatum*. FIC_D2_ refers to the fractional inhibitory concentration of diminazene aceturate. Three independent tests were performed after each combination was loaded in triplicate wells in 96-well plates.

## Data Availability

On reasonable request, the corresponding author will provide the datasets created and/or analyzed during the current work.

## Supplementary Materials

Supplementary 1 Table S1: viability test results of diminazene aceturate drug evaluated for *Babesia* and *Theileria* parasite.

Supplementary 2 Table S2: fluorescence values of the inhibitory effect of pomegranate (*Punica granatum*) peel mono- and combination therapies on the growth of *Babesia microti* in BALB/C mice.

Supplementary 3 Figure S1: PCR of the *ss-rRNA* gene in blood and different organs of *B. microti*-infected mice. A. Infected nontreated mice (positive control) and noninfected and nontreated mice (negative control). B. Mice treated with 25 mg kg^−1^ diminazene aceturate. C. Mice treated with pomegranate (*Punica granatum*) peel combined with diminazene aceturate. PC: positive control; NC: negative control; Bl: blood; Hr: heart; Lg: lung; Lv: liver; Kd: kidney; Sp: spleen. M indicates a 100 bp DNA ladder.
