# Supplementary material for: Pomegranate (Punica granatum) Peel Inhibits the In Vitro and In Vivo Growth of Piroplasm Parasites
Source: J Parasitol Res. 2022 Jun 20;2022:8574541. doi: 10.1155/2022/8574541 (PMC9237696; doi:10.1155/2022/8574541)
Supplement: Supplementary 1 — Table S1: viability test results of diminazene aceturate drug evaluated for Babesia and Theileria parasite. [file 8574541.f1.docx]

**Table S1. Viability test results of diminazene aceturate drug evaluated for *Babesia* and *Theileria* parasite**

| **Drug** | **Drug concentrations (μM) ^a^** | | | | |
| --- | --- | --- | --- | --- | --- |
|  | **10** | **5** | **1** | **0.5** | **0.25** |
| ***B. bovis*** | - | - | - | - | - |
| ***B. bigemina*** | - | - | - | - | - |
| ***B. divergens*** | - | - | - | - | - |
| ***T. equi*** | - | - | - | - | - |
| ***B. caballi*** | - | - | - | - | - |

a Each value was calculated using *Babesia* fluorescence assay (*B*FA) in three separate experiments. Each concentration of the drug was made in triplicate in each experiment. + = viable; − = dead
