# Supplementary material for: Pomegranate (Punica granatum) Peel Inhibits the In Vitro and In Vivo Growth of Piroplasm Parasites
Source: J Parasitol Res. 2022 Jun 20;2022:8574541. doi: 10.1155/2022/8574541 (PMC9237696; doi:10.1155/2022/8574541)
Supplement: Supplementary 2 — Table S2: fluorescence values of the inhibitory effect of pomegranate (Punica granatum) peel mono- and combination therapies on the growth of Babesia microti in BALB/C mice. [file 8574541.f2.docx]

Table S2. Fluorescence values of the Inhibitory effect of pomegranate (*Punica granatum)* peel mono- and combination therapies on the growth of *Babesia microti* in BALB/C mice

| **Days P.I.** | **DMSO control** | | **Diminazene aceturate 25 mg/kg** | | **Pomegranate (*Punica granatum)* peel 75 mg/kg** | | **Pomegranate (*Punica granatum)* peel 50 mg/kg + DA 15 mg/kg** | |
| --- | --- | --- | --- | --- | --- | --- | --- | --- |
|  | mean | std | mean | std | mean | std | mean | std |
| **0** | 0 | 0 | 0 | 0 | 0 | 0 | 0 | 0 |
| **2** | 0 | 0 | 0 | 0 | 0 | 0 | 0 | 0 |
| **4** | 43.78 | 23.71661 | 71.36 | 34.84409 | 157.94 | 36.34505 | 106.5265 | 7.0735 |
| **6** | 172.345 | 39.68365 | 134.34 | 71.9239 | 195.32 | 16.2 | 40.615 | 24.29488 |
| **8** | 1362.432 | 374.1895 | 188.895 | 111.6769 | 705.97 | 175.3671 | 228.345 | 112.4135 |
| **10** | 2044.96 | 176.697 | 700.76 | 97.22675 | 1538.61 | 346.1375 | 537.71 | 141.7045 |
| **12** | 1595.66 | 151.807 | 754.66 | 132.7168 | 1436.96 | 133.9636 | 535.735 | 304.3828 |
| **14** | 1086.48 | 174.0253 | 367.52 | 136.4687 | 1030.547 | 110.2822 | 300.455 | 142.4771 |
| **16** | 823 | 220.0787 | 150.42 | 112.0557 | 800.15 | 224.0574 | 216.975 | 103.7505 |
| **18** | 769.55 | 107.6927 | 1.54 | 3.443545 | 700.2 | 233.8712 | 37.1 | 18.55 |
| **20** | 486.8 | 90.8629 | 65.7 | 69.27666 | 219.325 | 96.6678 | 179.85 | 173.0455 |
| **22** | 315.625 | 46.9382 | 26.54 | 36.48353 | 307.45 | 176.8215 | 56.66667 | 98.14954 |
| **24** | 491.912 | 99.6515 | 45.7496 | 32.30909 | 326.2286 | 151.0303 | 62.712 | 11.80868 |
| **26** | 307 | 74.0276 | 82.2155 | 62.48 | 360.6625 | 38.5941 | 29.1625 | 11.1545 |
| **28** | 203.285 | 34.08 | 46.1 | 13.0827 | 106.75 | 31.6777 | 15.125 | 10.25 |
| **30** | 161.9925 | 42.3 | 7.3 | 6.3233 | 111.24 | 67.5464 | 9.588 | 3.66 |
