# Supplementary figures and images for: Pomegranate (Punica granatum) Peel Inhibits the In Vitro and In Vivo Growth of Piroplasm Parasites
Source: J Parasitol Res. 2022 Jun 20;2022:8574541. doi: 10.1155/2022/8574541 (PMC9237696; doi:10.1155/2022/8574541)

(A)

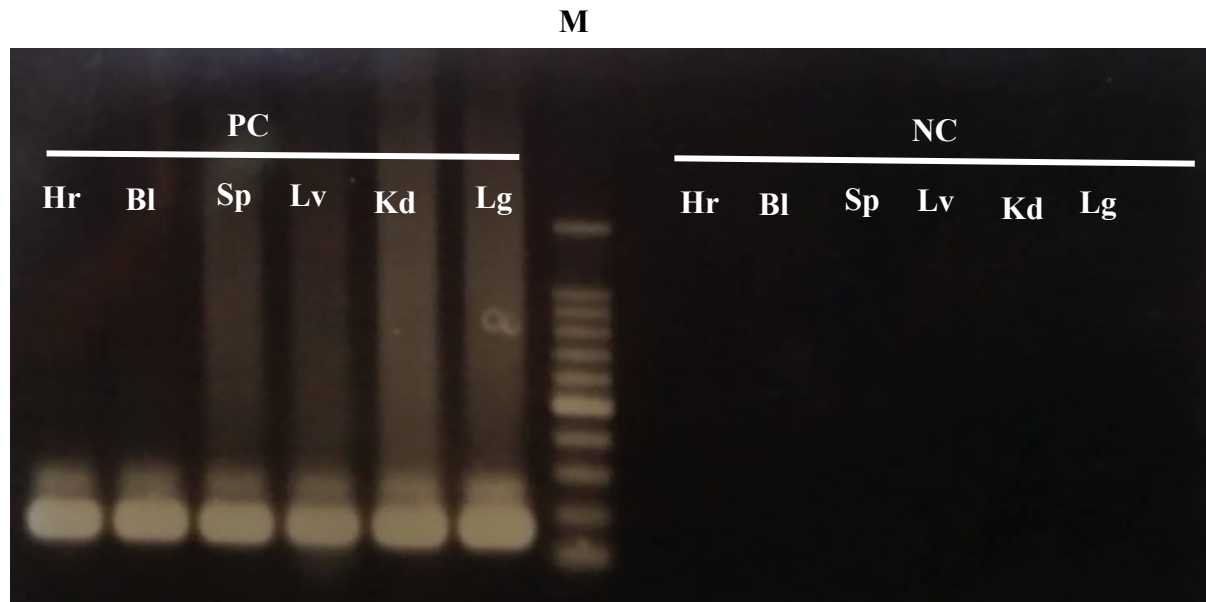

(B)

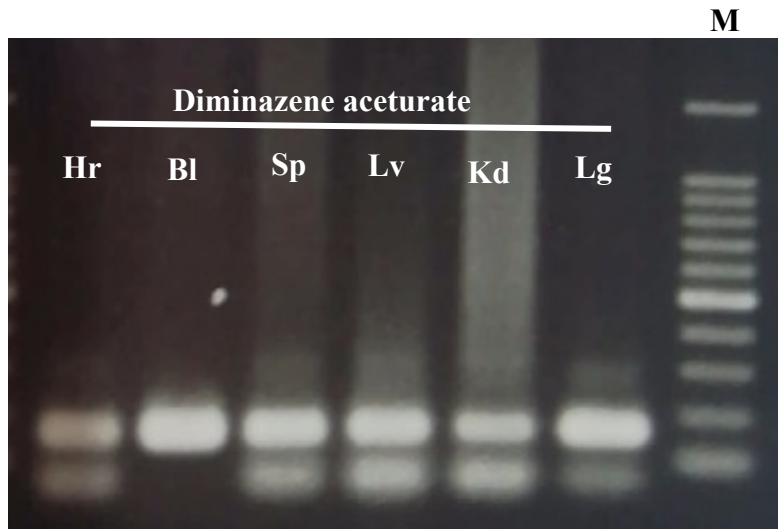

(C)

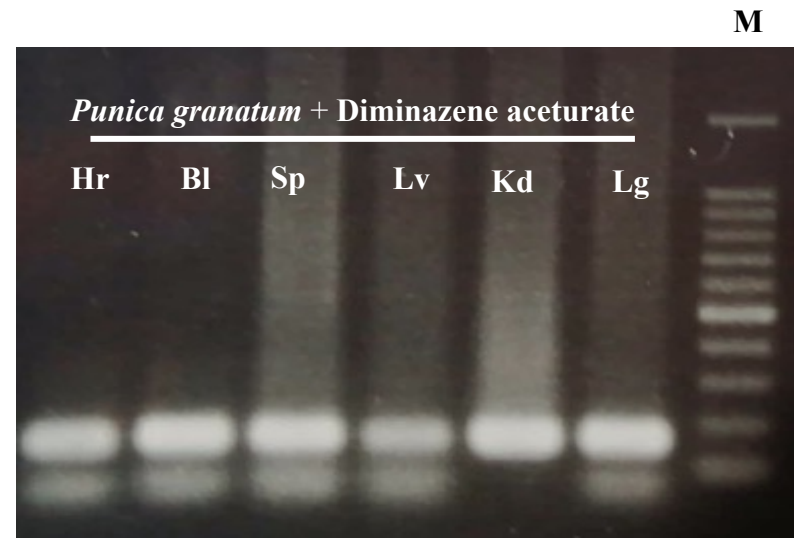

Supplement: Supplementary 3 — Figure S1: PCR of the ss-rRNA gene in blood and different organs of B. microti-infected mice. A. Infected nontreated mice (positive control) and noninfected and nontreated mice (negative control). B. Mice treated with 25 mg kg−1 diminazene aceturate. C. Mice treated with pomegranate (Punica granatum) peel combined with diminazene aceturate. PC: positive control; NC: negative control; Bl: blood; Hr: heart; Lg: lung; Lv: liver; Kd: kidney; Sp: spleen. M indicates a 100 bp DNA ladder. [file 8574541.f3.pdf]
